# Multi-omics analysis of lactate metabolism gene regulation in *Clonorchis sinensis*-associated hepatocellular carcinoma

**DOI:** 10.1186/s13071-025-06947-0

**Published:** 2025-07-27

**Authors:** Qiumei Lin, Junxian Chen, Lingling Zhou, Min Fang, Caibiao Wei, Taijun Huang, Yulong Xu, Jie Gao, Fengfei Liu, Zeli Tang, Jian-Kang Zhu, Weilong Yang

**Affiliations:** 1 Department of Clinical Laboratory, Guangxi Medical University Cancer Hospital, Nanning, China; 2 Guangzhou Women and Children’s Medical Center, Guangzhou Medical University, Guangzhou, China; 3 Institute of Advanced Biotechnology, and School of Medicine, Southern University of Science and Technology, Shenzhen, China; 4 Department of Cell Biology and Genetics, School of Basic Medical Sciences, Guangxi Medical University, Nanning, 530021 China; 5 Key Laboratory of Basic Research on Regional Diseases, Education Department of Guangxi Zhuang Autonomous Region, Guangxi Medical University, Nanning, China; 6 Engineering Research Center for Tissue & Organ Injury and Repair Medicine, Guangxi Medical University Cancer Hospital, Nanning, China

**Keywords:** *Clonorchis sinensis*, Hepatocellular carcinoma, Lactate metabolism, Multi-omics

## Abstract

**Background:**

Although recent research has highlighted lactylation, a post-translational modification driven by elevated lactate levels, as a critical regulator of key cellular pathways in hepatocellular carcinoma (HCC), its contribution to the poor prognosis of Clonorchis sinensis (Cs)-infected HCC remains poorly understood.

**Methods:**

We first identified the significant upregulation of the lactate metabolism enzyme LDH in Cs-infected HCC patients through clinical retrospective analysis. We then conducted a multi-omics analysis (RNA-Seq, ATAC-Seq, WGBS-Seq, oxWGBS-Seq, and ChIP-Seq) to examine the differences in 392 lactate metabolism-related genes (LMRGs) between Cs-infected and Cs-noninfected HCC tumors. Six key differentially expressed LMRGs were further validated using RT-qPCR assays to confirm their expression and potential role in HCC progression.

**Results:**

The differential expression levels of 8 LMRGs, along with 71 accessible regions and 42 CpG sites in the promoters of LMRGs, were identified. Notably, we also demonstrated that histone modifications, including H3K9ac, H3K79me2, H3K4me2, H3K4me3, H3K27ac, and H3K4me1, were associated with chromatin accessibility in the promoters of LMRGs. Finally, the TCGA-LIHC cohort confirmed that the differential expression of LMRGs between Cs-infected and Cs-noninfected HCC tumors significantly affects the survival outcomes of HCC.

**Conclusions:**

Our findings revealed that lactylation plays an important role in reshaping the characteristics of HCC during Cs infection, expanding our understanding of the unique features of Cs-infected HCC.

**Graphical Abstract:**

**Figure:**
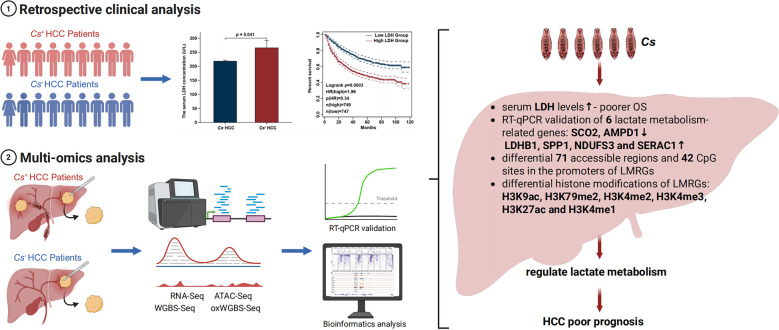

**Supplementary Information:**

The online version contains supplementary material available at 10.1186/s13071-025-06947-0.

## Background

Primary liver cancer is one of the most common malignant tumors worldwide, ranking sixth in global cancer incidence and third in cancer-related mortality [1, 2]. Among the various histological subtypes, hepatocellular carcinoma (HCC) is the most prevalent form of liver cancer, with a multifactorial pathogenesis involving chronic viral hepatitis, alcoholic liver disease, and parasitic infections [3, 4]. *Clonorchis sinensis* (*Cs*), a foodborne parasite endemic to East and Southeast Asia, is transmitted to humans through the ingestion of raw or undercooked freshwater fish or shrimp harboring metacercariae [5]. *Cs* infection is widely recognized as a major risk factor for HCC due to its ability to induce chronic inflammation, oxidative stress, angiogenesis, and the maintenance of cancer stem cell properties [6–9]. Despite growing evidence linking *Cs* infection to HCC, the precise molecular mechanisms, particularly those involving tumor metabolic regulation, remain inadequately understood. Therefore, elucidating these mechanisms is essential for advancing our understanding of *Cs*-associated HCC and developing targeted therapeutic strategies.

Tumor metabolism is characterized by a metabolic shift toward glycolysis, even in the presence of oxygen, a phenomenon known as the Warburg effect [10]. This metabolic reprogramming leads to lactate accumulation, with excess lactate secreted into the tumor microenvironment, where it promotes tumor progression by enhancing cell proliferation, immune evasion, and other key processes [9, 11]. For instance, lactate modulates immune cell functions to promote immune suppression; in breast cancer, it induces GPR81 expression in dendritic cells via paracrine signaling, impairing their antigen-presenting capabilities and fostering an immunosuppressive microenvironment [12, 13]. Beyond immune modulation, lactate-driven lactylation, a post-translational modification, has emerged as a key mechanism in drug resistance [14]. In gastric cancer, the upregulation of lactate dehydrogenase A (LDHA), a critical enzyme in lactate production, has been associated with resistance to neoadjuvant chemotherapy [15]. Lactate secreted by gastric cancer cells activates the NF-κB pathway, resulting in upregulated BDNF expression in cancer-associated fibroblasts, which contributes to drug resistance [16]. Furthermore, cancer-related lactylation at the K388 site of the NBS1 protein enhances DNA damage repair, thereby exacerbating chemotherapy resistance [15]. In HCC, protein lactylation emerges as a central regulator of tumor progression; it orchestrates tumor metabolism, facilitates invasion and metastasis, and reinforces resistance to both chemotherapy and immunotherapy [8, 17]. For instance, lactylation at the K28 site of adenylate kinase 2 inhibits its kinase activity, leading to intracellular energy imbalance and promoting the proliferation, invasion, and metastasis of HCC cells [18]. Additionally, lactylation at H3K56 and ALDOA K230/322 sites enhances the stemness properties of liver cancer stem cells, thereby driving the initiation and progression of HCC [19].

By systematically screening key lactate metabolism-related genes (LMRGs) and the potential role of lactylation in driving cancer progression through its regulation of immune responses and tumor metabolism, this modification may offer valuable molecular insights into the mechanisms underlying poor prognosis in *Cs*-associated HCC. In this study, we performed a retrospective analysis of clinical data to identify differences in lactate dehydrogenase (LDH) levels between *Cs*-infected (*Cs*^+^) and *Cs*-noninfected (*Cs*^−^) HCC, integrating multi-omics data, including RNA-seq, ATAC-seq, ChIP-seq, and WGBS-seq. We comprehensively investigated the alterations in lactate-related gene expression, chromatin accessibility, histone modifications, and DNA methylation patterns in *Cs*^+^ HCC tumors compared to *Cs*^−^ HCC tumors. Furthermore, we identified specific lactate-related genes whose expression changes are significantly associated with poor prognosis in *Cs*^+^ HCC, providing critical insights into the molecular mechanisms that may underlie the aggressive progression and poor clinical outcomes of this disease.

## Methods

### Samples collection

Liver tissue samples were obtained from HCC patients who had not received any prior anti-cancer treatments and underwent surgical resection at the Department of Hepatobiliary Surgery, Guangxi Medical University Cancer Hospital (Nanning, China). All patients were treatment-naive at the time of surgery.

Tissue specimens displaying typical macroscopic characteristics were taken from the tumor nodules and were subsequently analyzed histopathologically. The tissue sections were stained with hematoxylin and eosin (H&E) for confirmation of HCC diagnosis. In addition, paired adjacent non-cancerous liver tissues were collected from regions located at least 5 cm away from the tumor margin. The study was approved by the Guangxi Medical University Cancer Hospital Ethical Review Committee (KY2025016) and conducted in accordance with the Declaration of Helsinki. Upon admission, all patients provided written consent for the analysis and publication of their anonymized medical data for research purposes.

### Study population and data collection

A retrospective review was conducted to assess the levels of lactate dehydrogenase (LDH) in 1496 HCC patients who underwent surgical treatment between 2013 and 2022. The inclusion criteria for this study were as follows: (i) a confirmed diagnosis of HCC based on postoperative pathology after surgical resection; (ii) no prior treatment with anti-cancer therapies; (iii) no history of other malignancies; (iv) availability of complete laboratory and pathological data.

*Clonorchiasis* was diagnosed through one or more of the following methods: (i) preoperative imaging (MRI, CT, microscopy, or ultrasound) showing the presence of *Cs* eggs or adult worms in the intrahepatic bile ducts, (ii) identification of adult *Cs* in the liver or gallbladder during intraoperative or postoperative pathology, or (iii) preoperative fecal examination revealing *Cs* eggs.

For postoperative metastatic surveillance, patients underwent regular imaging (CT, MRI, or PET) and clinical assessments to monitor for potential metastasis, including spread to extrahepatic sites (e.g. lungs, bones, lymph nodes) or other parts of the liver. Metastatic lesions were confirmed through tissue biopsy, which included intra- or postoperative pathological examination of resected specimens to detect tumor cells in distant tissues.

### Collection of the lactylation metabolism-related gene set

The lactylation metabolism-related genes (LMRGs) were collected from the Molecular Signatures Database [20]. Finally, 392 LMRGs were obtained.

### Analysis of RNA-seq data

Raw sequencing data were processed to eliminate low-quality reads and adapter sequences using Trim Galore (v.0.6.10) [21]. The filtered high-quality reads were aligned to the hg38 reference genome using Hisat2 (v.2.2.1) [22] with default parameters. A gene expression matrix was subsequently constructed using featureCounts (v.2.0.6) [23]. To assess differential gene expression, we used the R package 'DESeq2' (v.1.44.0) [24], applying a threshold of |Fold Change|> 2 and p < 0.05. Gene Ontology (GO) and Kyoto Encyclopedia of Genes and Genomes (KEGG) enrichment analyses were performed with the 'clusterProfiler' R package (v.4.12.0) [25] to investigate the biological processes and pathways associated with the differentially expressed genes.

### Analysis of ATAC-Seq data

Raw sequencing data were processed to remove low-quality reads and adapter sequences using Trim Galore (v.0.6.10) [21], resulting in high-quality data. These clean reads were then aligned to the human hg38 reference genome using Bowtie2 (v.2.5.1) [26] with the following parameters: –very-sensitive -X 2000. PCR duplicates were removed using Sambamba (v.0.6.6) [27], and mitochondrial DNA sequences were excluded from the analysis. The resulting alignment BAM files were converted into bigwig files using DeepTools bamCoverage with the– normalizedUsing RPKM option. Peaks were identified with MACS2 using the parameters -g hs– nomodel– shift-100– extsize 200. Peak visualization was carried out with the Integrative Genomics Viewer (IGV) (v.2.16.1) [28]. Differentially accessible peaks were detected using the R package ‘DESeq2’ (v.1.44.0) [24], applying a threshold of |Fold Change|> 2 and *p* < 0.05. Motif enrichment analysis was performed using the HOMER tool (findMotifsGenome.pl) with default parameters (*p* < 0.01). Peak annotation was conducted with the annotatePeak function from the R package ‘ChIPseeker’ (v.1.34.1) [29]. Promoter regions were defined as the regions within 3 kb upstream and downstream of the transcription start site (TSS). Gene activity scores were calculated by counting the number of reads in peaks associated with promoter regions for each gene. The parameters for identifying differentially expressed genes (DEGs) and gene activity scores were |log2(Fold Change)|> 0.5 and *p* < 0.05.

### Integrative analysis of ChIP‑seq and ATAC‑seq data

Due to the extreme scarcity of ChIP-seq data from primary HCC samples, particularly those with *Cs* infection, we utilized these well-characterized HepG2 datasets as the most currently available alternative. Bigwig files from the chromatin immunoprecipitation followed by sequencing (ChIP-seq) analysis of HepG2 cells (GSE29611) were obtained from the GEO database. Genome coordinate conversion from hg19 to hg38 was performed using CrossMap (v.0.7.0) [30]. Enrichment signals from both ChIP-seq and assay for transposase-accessible chromatin using sequencing (ATAC-seq) were analyzed with the computeMatrix tool from DeepTools, and the enrichment patterns were visualized using the plotHeatmap function in DeepTools. Representative regions of interest were displayed using the Integrative Genomics Viewer (IGV) (v.2.16.1) [28].

### WGBS and oxWGBS data processing

Trim Galore (v.0.6.10) [31] was used to trim low-quality reads and remove adapter sequences from the raw sequencing data, generating high-quality reads. The cleaned reads were aligned to the hg38 reference genome using BSMAP (v.2.90) [32] with the following parameters: -p 10 -v 0.05. PCR duplicates were eliminated using Sambamba (v.0.6.6) [27]. Methylation levels at each CpG site were extracted using the Python-based script methratio.py, which is included in BSMAP. Only CpG sites with a minimum coverage of 10 reads were included in the subsequent analysis. Differentially methylated sites were identified using the R package 'limma' (v.3.54.2), with |logFC|≥ 0.5 and *p* < 0.05 as the criteria for identifying significant methylation differences.

### RT-qPCR

Total ribonucleic acid (RNA) was extracted from the tumor tissues of *Cs*^+^ HCC and *Cs*^−^ HCC patients. The tissue samples were first ground into a fine powder in liquid nitrogen, and RNA was extracted using Trizol reagent (Invitrogen, USA) following the manufacturer’s protocol. Then, 1 μg of RNA was reverse transcribed into complementary DNA (cDNA) using the Reverse Transcription Master Kit (Takara, Japan) according to the manufacturer’s instructions. Reverse transcription-quantitative PCR (RT-qPCR) was performed using the qTOWER3 Fluorescence Quantitative PCR Instrument (Jena, Germany) and TB Green Premix Ex Taq II FAST qPCR (2X) (Takara, Japan). Relative gene expression levels were normalized to U6 miRNA and calculated using the 2^-ΔΔCt method. The experiments were repeated three times to ensure the consistency and reliability of the results. Bulge-loop RT primers and qPCR primers specific for LDHB, SPP1, NDUFS3, SERAC1, SCO2, and AMPD1 were designed and synthesized by Sangon Biotech (Shanghai, China). The primers used in this study are listed in Table S6. Primer specificity was assessed using the Basic Local Alignment Search Tool (BLAST) available at the National Center for Biotechnology Information (NCBI) website (https://www.ncbi.nlm.nih.gov/).

### Survival analysis

We performed survival analysis using the Gene Expression Profiling Interactive Analysis 2 (GEPIA2) website (http://gepia2.cancer-pku.cn/#survival), based on the The Cancer Genome Atlas—Liver Hepatocellular Carcinoma (TCGA-LIHC) cohort with default parameters. A *p*-value < 0.05 indicates that the mRNAs are associated with the survival prognosis of HCC patients.

### TCGA database analysis

Univariate Cox regression analysis was done by the R package ‘survival’ v.3.8–3 and ‘survminer’ v0.5.3 based on the TCGA-LIHC cohort. The R package ‘TCGAplot’ (v.7.0.1) was used to do the analysis, and the TCGA-LIHC cohort was performed to analyze HCC. In detail, gene_methylation_scatter was used to calculate the relationship between gene expression and gene promoter methylation; methy_kmplot was used to do survival analysis of the promoter methylation of a specific gene; tcga_kmplot was used to do survival analysis of a specific gene; gene_network_go was used to create a cnetplot to depict the linkages of gene(s) and GO terms as a network; gene_gene_scatter was used to draw scatter plot of gene and gene correlation in a specific type cancer; gene_coexp_heatmap was used to draw heatmap and Go enrichment of the positive and negative co-expressed genes of a single gene in a specific type of cancer.

### Statistical analysis

Statistical analysis was performed using R software (v.4.3.0). Pearson’s correlation test was used to assess the relationships between variables, with statistical significance defined as *p* < 0.05. All statistical analyses were conducted with GraphPad Prism version 8.0. Data are expressed as means ± standard deviation (SD) from three independent experiments. Differences between groups for categorical variables (expressed as ratios) were compared using the chi-square test. A *p*-value < 0.05 was considered statistically significant (*p < 0.05).

### Data availability

The raw sequencing data generated in this study, including RNA-Seq, WGBS-Seq, oxWGBS-Seq, and ATAC-Seq, have been deposited in public repositories. RNA-Seq data are available in the NCBI Sequence Read Archive under BioProject accession no. PRJNA1173109. WGBS-Seq and oxWGBS-Seq data have been deposited in both the NCBI Sequence Read Archive and the GEO database (accession no. GSE284332). ATAC-Seq data are available in the GEO database under accession no. GSE276855. Additionally, publicly available ChIP-Seq datasets used in this study were obtained from the GEO repository (accession no. GSE29611).

## Result

**Fig. 1:**
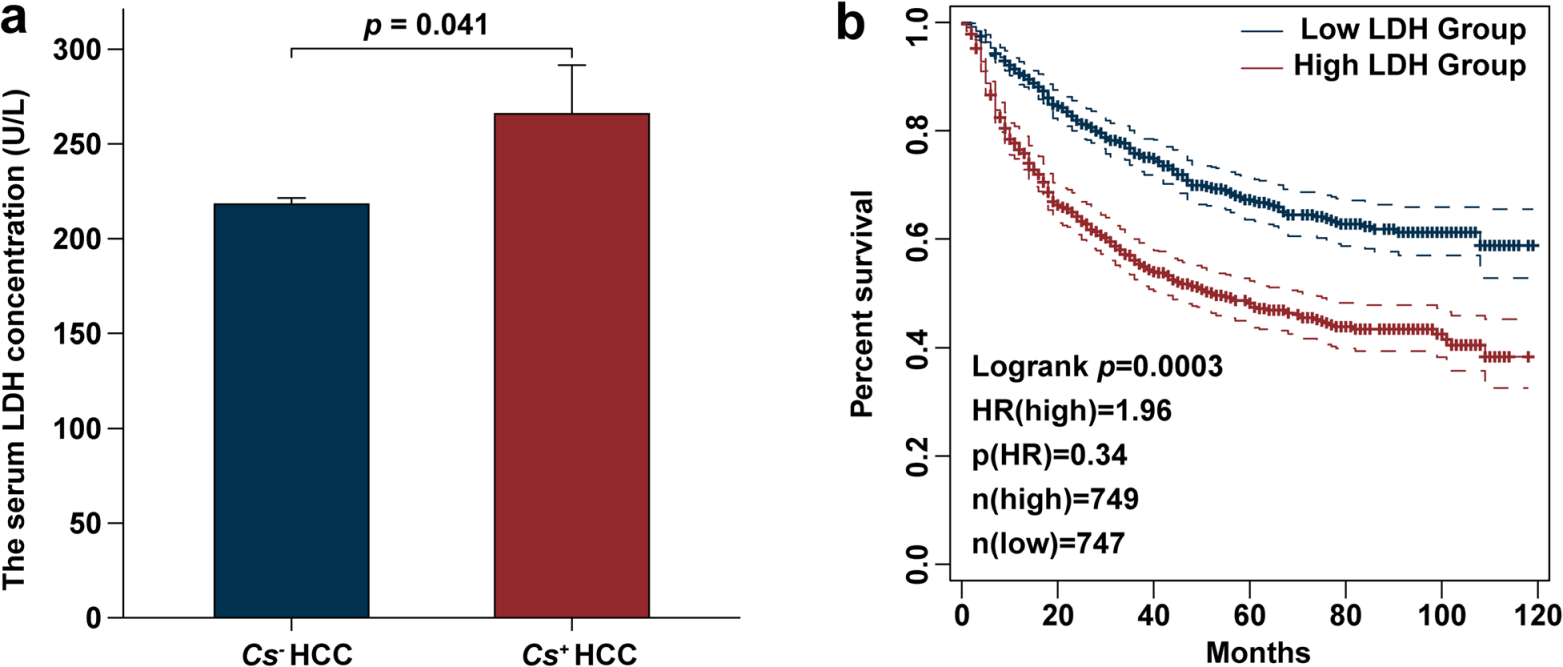
Retrospective clinical analysis of the serum key enzyme LDH in lactate metabolism among *Cs*^+^ and *Cs*^−^ HCC patients. **a** Comparative analysis of serum LDH concentrations between *Cs*^+^ and *Cs*^−^ HCC patients. **b** Kaplan-Meier survival curves demonstrating the correlation between stratified serum LDH levels and overall survival outcomes

We first investigated the differences in lactate dehydrogenase (LDH) levels, a key clinical marker of lactate metabolism and a well-established biomarker for metabolic reprogramming in cancer, between *Cs*^+^ and *Cs*^−^ HCC patients [33]. Through a retrospective analysis of LDH concentrations in 1496 HCC patients from 2013 to 2022, our results revealed that serum LDH levels were significantly higher in *Cs*^+^ HCC patients compared to *Cs*^−^ HCC patients (Fig. 1a). The information on all patients was listed in Table S1. Furthermore, in this retrospective cohort, elevated serum LDH concentrations were strongly associated with poorer overall survival outcomes (Fig. 1b), highlighting its potential as a prognostic marker for poor HCC prognosis driven by *Cs* infection.

### RNA-seq analysis of lactate metabolism-related genes (LMRGs) between *Cs*^+^ and *Cs*^−^ HCC

**Fig. 2:**
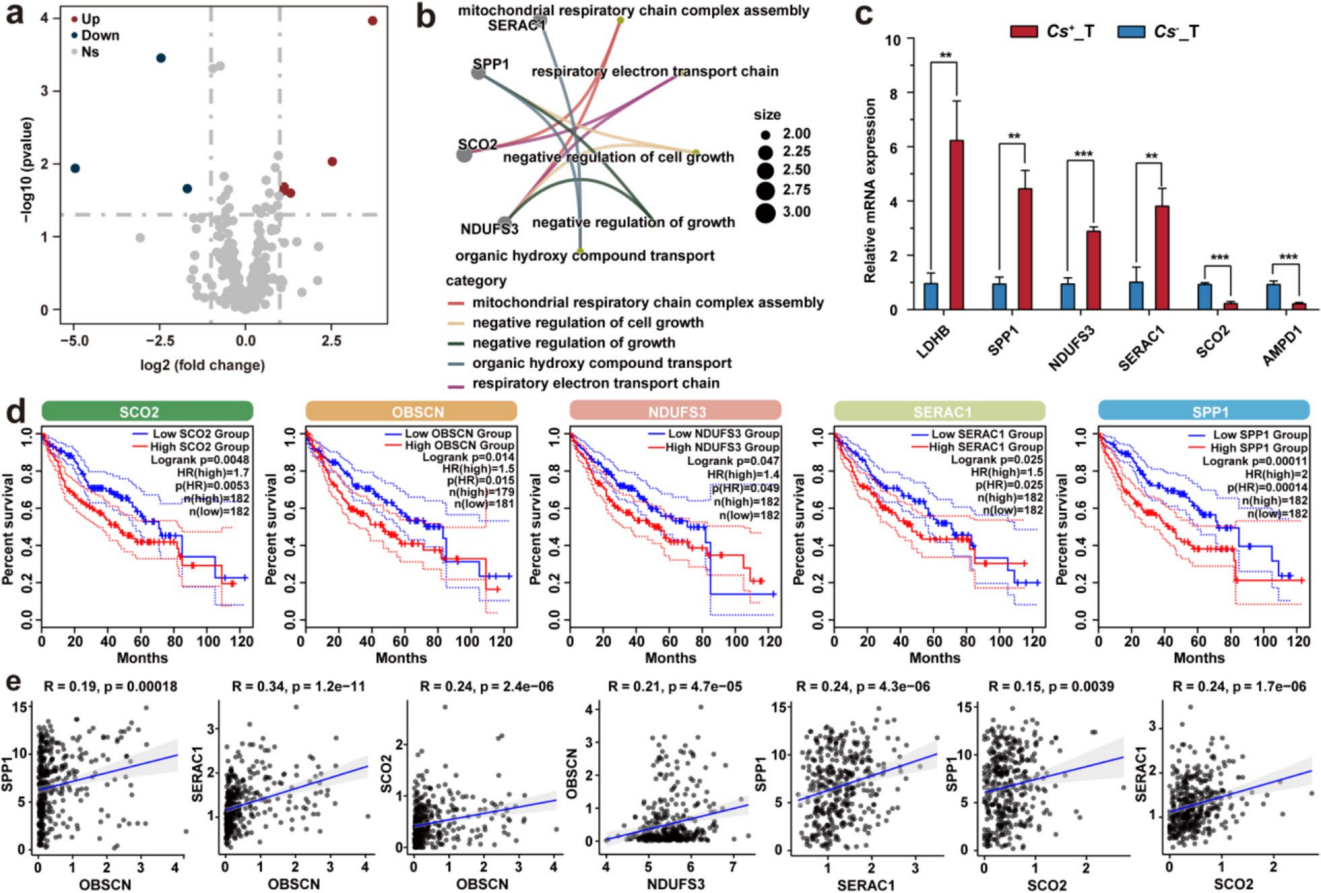
*Cs* infection changes the expression profile of LMRGs in HCC tumors. **a** Volcano plot showing downregulated and upregulated LMRGs between *Cs*^+^ HCC tumors and *Cs*^−^ HCC tumors. **b** GO enrichment network of differentially expressed LMRGs between *Cs*^+^ HCC tumors and *Cs*^−^ HCC tumors. **c** RT-qPCR validation of differentially expressed LMRGs in *Cs*^+^ and *Cs*^−^ HCC tumor samples. Data were presented as means ± SD; *n* = 3. Student’s t-test was used. **d** Kaplan-Meier curves illustrating the association between the expression levels of differentially expressed LMRGs and survival outcomes in the TCGA-LIHC cohort. **e** Spearman correlation analysis assessing the relationships among differentially expressed LMRGs

**Fig. 3:**
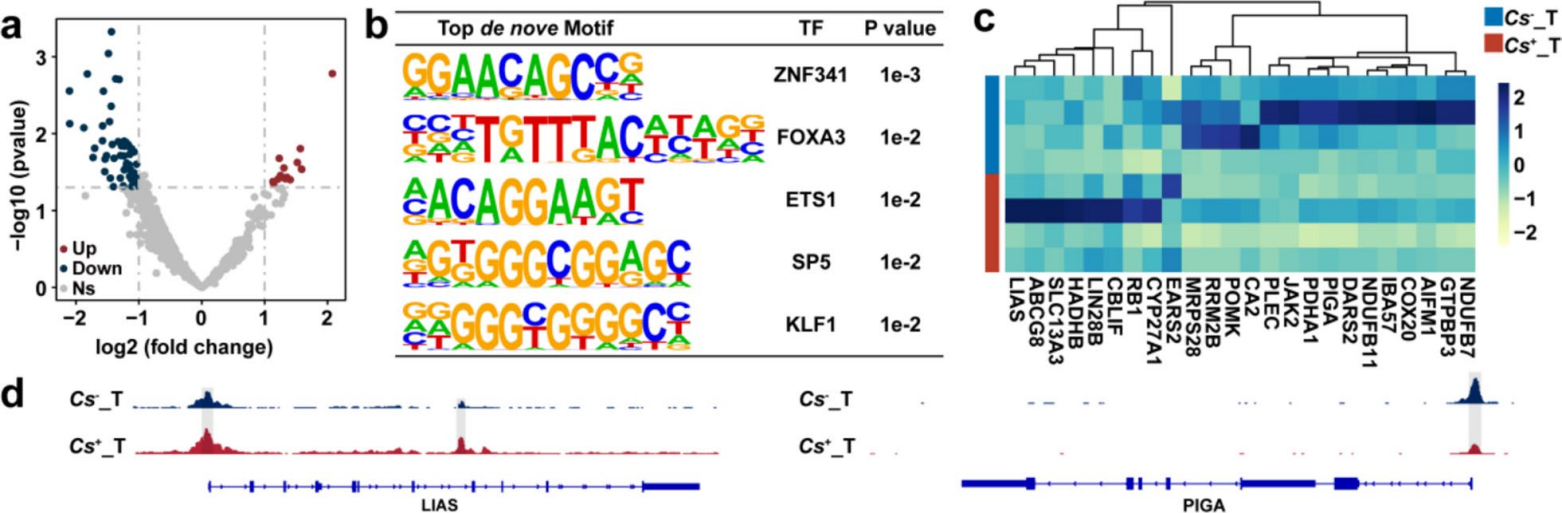
*Cs* infection changes the chromatin accessibility landscape of lactate metabolism-related genes in HCC tumors. **a** Different chromatin accessibility landscapes in the promoter regions of LMRGs between *Cs*^+^ HCC tumors and *Cs*^−^ HCC tumors. **b** The top de novo motifs enriched in differential chromatin accessibility regions in the promoter regions of LMRGs between *Cs*^+^ HCC tumors and *Cs*^−^ HCC tumors. **c** Heatmap representation of gene active scores in differential chromatin accessibility regions of LMRGs between *Cs*^+^ HCC tumors and *Cs*^−^ HCC tumors. **d** IGV shows representative difference peaks of LMRGs between *Cs*^+^ HCC tumors and tumor-adjacent tissues

Considering the elevated serum LDH concentrations in *Cs*^+^ HCC patients, we sought to further elucidate the molecular mechanisms driving changes in lactate metabolism. To explore the changes in lactate metabolism induced by *Cs* infection and their role in cancer progression, we curated a list of lactate metabolism-related genes (LMRGs) and compared the expression of these genes in 10 *Cs*^+^ HCC tumors and 10 *Cs*^−^ HCC tumors, obtained from our previous study. Through differential gene screening, we identified eight differentially expressed LMRGs, comprising three downregulated and five upregulated genes (Table S2). These differentially expressed LMRGs were visualized in a volcano plot (Fig. 2a). GO enrichment analysis revealed that these genes were involved in processes such as mitochondrial respiratory chain complex assembly, negative regulation of cell growth, negative regulation of growth, organic hydroxy compound transport, and respiratory electron transport chain (Fig. 2b). To validate the eight differentially expressed LMRGs, we performed RT-qPCR analysis on *Cs*^+^ and *Cs*^−^ HCC tumor samples. The results indicated that, compared to *Cs*^−^ HCC, the expression levels of LDHB, SPP1, NDUFS3, and SERAC1 were significantly upregulated in *Cs*^+^ HCC. Conversely, SCO2 and AMPD1 showed significant downregulation in *Cs*^+^ HCC compared to *Cs*^−^ HCC (Fig. 2c). Overall, the RT-qPCR results were consistent with our RNA-seq findings. Additionally, we performed prognostic analysis on these differentially expressed LMRGs using the TCGA-LIHC cohort and found that the upregulation of SCO2, OBSCN, NDUFS3, SERAC1, and SPP1 was significantly associated with poor prognosis (Fig. 3d). Although the expression level of LDHB was not significantly correlated with overall survival, its upregulation was still associated with poorer survival, as shown in the survival curve (Fig. S1a). Furthermore, we conducted a correlation analysis of these five prognosis-related genes and found that their expression levels were generally significantly positively correlated (Fig. 2e), except NDUFS3, whose expression showed no significant correlation with that of SPP1, SERAC1, or SCO2 (Fig. S1b). Additionally, we also performed co-expression analysis on SCO2, OBSCN, NDUFS3, SERAC1, and SPP1 (Fig. S2–S6). Finally, we conducted a stratified analysis of LMRG expression across clinical tumor stages (BCLC-B vs. BCLC-A). Initial analyses identified significant upregulation of four LMRGs, including SLC4A4, in advanced-stage (BCLC-B) *Cs*^+^ HCC tumors compared to the early-stage (BCLC-A). This stage-dependent expression pattern suggests a potential role for these metabolic regulators in HCC progression (Table S7).

### ATAC-seq profiling of chromatin accessibility in LMRGs between *Cs*^+^ and *Cs*^−^ HCC

Given the important role of lactate in epigenetic regulation, we further analyzed chromatin accessibility related to the promoters of LMRGs in 4 *Cs*^+^ HCC tumors and 4 *Cs*^−^ HCC tumors using ATAC-seq data of our previous study. Compared to *Cs*^−^ HCC tumors, 14 regions exhibited positive enrichment, while 57 regions showed negative enrichment in *Cs*^+^ HCC tumors (Fig. 3a and Table S3). A de novo motif enrichment analysis of these regions delineated the top five most prominently enriched motifs as ZNF341, FOXA3, ETS1, SP5, and KLF1 (Fig. 3b). We also calculated activation scores for genes associated with differentially accessible promoter elements between *Cs*^+^ HCC tumors and *Cs*^−^ HCC tumors (Table S4). Based on these scores, we identified 24 differentially expressed genes, which are displayed in a heatmap (Fig. 3c). Finally, representative chromatin accessibility regions associated with LMRGs were also presented (Fig. 3d).

### ChIP-seq analysis of histone modifications in LMRGs between *Cs*^+^ and *Cs*^−^ HCC using public data

**Fig. 4:**
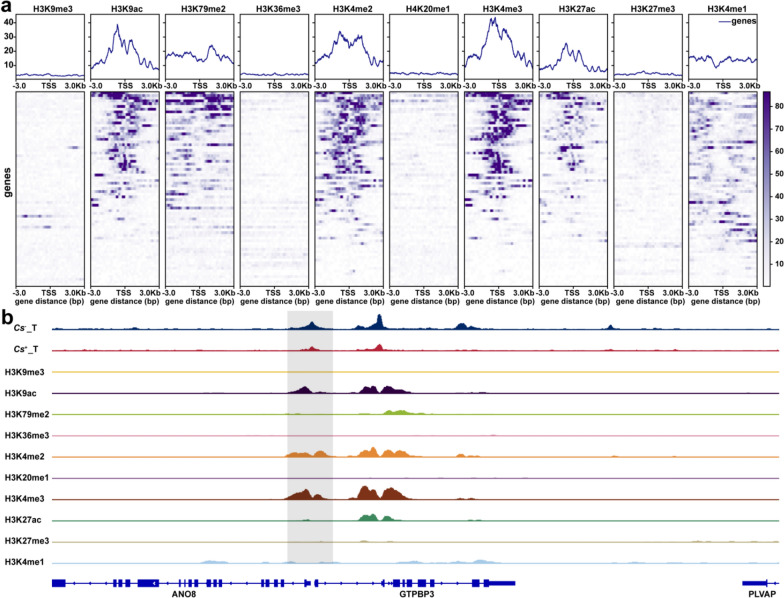
Joint analysis of ATAC-Seq and public ChIP-seq data between *Cs*^+^ HCC tumors and *Cs*^−^ HCC tumors. **a** Heatmap representation of histone modifications associated with differential chromatin accessibility regions of LMRGs between *Cs*^+^ HCC tumors and *Cs*^−^ HCC tumors. **b** IGV shows representative ATAC-seq and ChIP-seq signals between *Cs*^+^ HCC tumors and *Cs*^−^ HCC tumors

Histone modifications have been widely recognized for their role in regulating chromatin accessibility [34]. To examine how these modifications influence chromatin accessibility in the context of lactate metabolism changes caused by *Cs* infection, we accessed ChIP-seq data from the HepG2 cell line available in public repositories. This dataset includes key modifications such as H3K9me3, H3K9ac, H3K79me2, H3K36me3, H3K4me2, H4K20me1, H3K4me3, H3K27ac, H3K27me3, and H3K4me1. The heatmap displayed the enrichment signals of all ChIP-seq data in lactate metabolism-related differentially accessible regions between *Cs*^+^ HCC tumors and *Cs*^−^ HCC tumors. Notably, we observed that H3K9ac, H3K79me2, H3K4me2, H3K4me3, H3K27ac, and H3K4me1 were closely associated with the differential chromatin accessibility regions of LMRGs between *Cs*^+^ HCC tumors and *Cs*^−^ HCC tumors (Fig. 4a). We further visualized the ATAC-seq and ChIP-seq signals in selected representative regions (Fig. 4b).

### Whole-genome bisulfite sequencing (WGBS) and whole-genome oxidative bisulfite sequencing (oxWGBS) reveal DNA methylation of LMRGs between *Cs*^+^ and *Cs*^−^ HCC

**Fig. 5:**
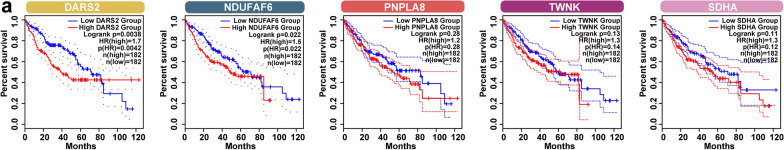
Correlation between DNA methylation of LMRGs and survival outcomes in the TCGA-LIHC cohort. **a** Kaplan-Meier curves show survival outcomes of genes associated with differential methylation sites in LMRGs between *Cs*^+^ HCC tumors and *Cs*^−^ HCC tumors

To further elucidate the lactate metabolism-related epigenetic changes induced by *Cs* infection, we analyzed DNA methylation and hydroxymethylation data obtained through WGBS and oxWGBS in four *Cs*^+^ HCC tumors and four *Cs*^−^ HCC tumors from our previous study. By comparing differentially methylated CpG sites in the promoter regions between *Cs*^+^ and *Cs*^−^ HCC tumors, we further identified differentially methylated promoter CpG sites associated with LMRGs (Table S5). However, no significant differences were found in the hydroxymethylation of promoter CpG sites associated with LMRGs. In subsequent analyses, we performed prognostic evaluation of the genes linked to these differentially methylated promoter CpG sites. Among the LMRGs associated with differentially methylated promoter CpG sites between *Cs*^+^ and *Cs*^−^ HCC tumors, six LMRGs associated with differentially methylated promoter CpG sites were identified: OBSCN, DARS2, NDUFAF6, PNPLA8, TWNK, and SDHA. Notably, high expression levels of OBSCN, DARS2, and NDUFAF6 were significantly correlated with poor overall survival, suggesting that *Cs* infection may drive changes in lactate metabolism-related pathways through DNA methylation, ultimately contributing to unfavorable HCC prognosis (Fig. 5a).

## Discussion

Alterations in lactate metabolism play a central role in cancer progression, not only driving tumor growth but also reshaping the tumor microenvironment by fostering immunosuppression and promoting angiogenesis [35, 36]. Lactate dehydrogenase (LDH), a critical enzyme in lactate metabolism, is well established as a biomarker for metabolic reprogramming in cancer [33]. Our retrospective clinical analysis revealed that serum LDH concentrations were significantly elevated in *Cs*^+^ HCC patients compared to *Cs*^*−*^ HCC patients, and high LDH levels were strongly associated with poor overall survival outcomes, underscoring their potential as a prognostic marker for *Cs*-driven HCC progression. These findings are consistent with previous studies identifying LDH as a key driver of tumor growth, angiogenesis, and immune evasion through lactate-dependent metabolic reprogramming in the tumor microenvironment, suggesting that *Cs* infection may promote tumor progression by upregulating LDH [8]. Transcriptomic analysis identified significantly upregulated LMRGs between *Cs*^+^ and *Cs*^*−*^ HCC tumors, including NDUFS3, SERAC1, SPP1, and LDHB, which were strongly associated with poor prognosis. Their differential upregulation was further validated by RT-qPCR. Notably, SPP1 has been widely reported to be associated with macrophage infiltration, cancer-associated fibroblasts, poor prognosis in hepatocellular carcinoma, and resistance to sorafenib and lenvatinib [37–39]. Additionally, chromatin accessibility analysis further revealed that *Cs* infection altered the lactate metabolism-related chromatin landscape in HCC. ZNF341, FOXA3, ETS1, SP5, and KLF1 binding motifs were enriched in differentially accessible regions associated with lactate metabolism between *Cs*^+^ and *Cs*^*−*^ HCC tumors. FOXA3, for instance, has been implicated in HCC metastasis by regulating PKM2 expression, with its activity modulated by the HDAC2/FOXA3 pathway [40, 41]. ETS-1 upregulation drives HCC progression by promoting MMP-7 overexpression and epigenetic silencing while also contributing to sorafenib resistance through the regulation of ferroptosis [42–44]. Beyond transcriptional regulation, our study emphasizes the significance of epigenetic modifications, such as histone modifications, in modulating chromatin structure and controlling gene expression [45]. Lactate plays a crucial role in epigenetic regulation, with some studies indicating that histone lysine lactylation is involved in regulating gene transcription [46, 47]. To further investigate these mechanisms, ChIP-seq data revealed that histone modifications, including H3K9ac, H3K79me2, H3K4me2, H3K4me3, H3K27ac, and H3K4me1, were enriched in differential chromatin accessibility regions associated with LMRGs. Notably, H3K27ac, H3K4me2, H3K4me3, and H3K9ac are key markers of active enhancers and promoters [48–50]. These modifications suggest that *Cs* infection may accelerate HCC progression by activating oncogenes and lactate metabolism genes, potentially through histone lactylation, which could modulate the epigenetic regulation of these genes, thereby driving tumor growth and metastasis, as observed in other cancer models [51, 52]. Finally, methylation analysis revealed that LMRGs, including OBSCN, DARS2, and NDUFAF6, which are associated with differentially methylated CpG sites in their promoters, are significantly linked to poor prognosis. This suggests that *Cs* may regulate lactate metabolism through DNA methylation, thereby contributing to a worse prognosis. Considering the relationship between *Cs* infection and lactate metabolism, LDH inhibitors or metabolic modulators targeting lactate may be beneficial in prolonging the survival of patients with *Cs*^+^ HCC, which holds significant potential for guiding relevant treatments.

Although we observed correlations among lactate metabolism, histone modifications, and DNA methylation, the direct mechanistic link remains to be clarified. Our interpretations regarding histone lactylation are based on associations with LDH activity and chromatin accessibility rather than direct measurement. Future studies should incorporate lactylome profiling, ChIP-seq with lactylation-specific antibodies, and metabolic isotope tracing to establish causality and map lactate-responsive regulatory regions. In addition to its impact on lactate metabolism, *Cs* infection appears to dysregulate multiple metabolic pathways, including fatty acid metabolism [53, 54]. The resulting metabolic perturbations may drive extensive crosstalk among glycolysis, TCA cycle intermediates, and lipid/amino acid metabolism, collectively reshaping the epigenetic landscape and promoting malignant progression. To fully elucidate this metabolic-epigenetic axis in *Cs*-induced HCC, an integrated approach combining multi-omics profiling (epigenomics, proteomics, metabolomics) with stable isotope-resolved metabolic flux analysis will be critical.

HCCs arising from viral (HBV/HCV) and alcoholic etiologies are well known to undergo Warburg-like metabolic reprogramming, characterized by increased glycolytic flux and lactate accumulation. Multiple studies and reviews have reported the consistent upregulation of glucose transporters (GLUT1/2) and glycolytic enzymes (HK2, GAPDH, PKM2, LDHA) across non-*Cs* HCC types [55]. In HBV-related HCC, viral proteins (e.g. HBx) have been shown to activate transcription factors such as NAC1, which drive LDHA expression and promote lactate production, immune escape, and tumor progression [56, 57]. Furthermore, analysis of the TCGA-LIHC dataset has identified lactate-related gene signatures—including SPP1, SLC16A3 (MCT4), and LDHD—that correlate with poor prognosis and aggressive phenotypes [58]. In alcoholic liver disease, LDH family enzymes are also upregulated under chronic inflammation and hypoxia; notably, LDHB knockdown alleviated ethanol-induced liver injury, linking lactate metabolism to alcohol-related HCC [55]. In contrast, our study highlights that in *Cs*-associated HCC, lactate metabolism is not only elevated (e.g. upregulation of LDH, SPP1) but also coupled with distinct epigenetic remodeling, including increased chromatin accessibility, and dynamic promoter methylation patterns. While LDH upregulation and glycolysis are shared across HCC subtypes, our multi-omics data suggest that *Cs* infection may accelerate or uniquely amplify the metabolic-epigenetic axis, likely due to parasite-specific immune microenvironments and sustained exposure to excretory-secretory products (*Cs*ESPs).

Parasites generally promote tumor progression by stimulating inflammation, shaping the immunosuppressive microenvironment, fibrosis, and epigenetic regulation, such as parasite-associated cancers. *Schistosoma haematobium* primarily drives bladder carcinogenesis through egg deposition, which induces chronic inflammation, fibrosis, and genomic instability, reshaping the local immune microenvironment [59–64]. Our discovery that *Cs* infection exacerbates HCC progression through lactic acid metabolic reprogramming provides novel insights into parasite-mediated oncogenesis. This mechanism partially resembles the metabolic shift toward inflammation-driven aerobic glycolysis (increased lactate production) also seen in HBV/HCV-related HCC [65, 66]. However, *Cs* uniquely exploits lactate to remodel DNA methylation and chromatin states, suggesting parasite-specific therapeutic targets. At the therapeutic level, combining LDH inhibitors (e.g. oxamate) with epigenetic drugs (e.g. DNMT inhibitors) may block *Cs*^+^ HCC progression, although challenges such as persistent parasitic infection require further investigation. Future studies should validate these targets in *Cs*-infected organoid models or patient-derived xenograft models to bridge mechanistic discoveries with clinical translation.

To the best of our knowledge, this is the first study to reveal, through clinical retrospective analysis and multi-omics approaches, that *Cs* infection leads to poor prognosis in HCC by regulating lactate metabolism. Our findings suggest that *Cs* infection induces profound changes in lactate metabolism, contributing to metabolic reprogramming and altered gene expression. However, several limitations must be considered. The generalizability of our findings may be constrained by the relatively limited sample size. Further validation through multicenter studies with expanded cohorts is needed, particularly incorporating stratified analyses based on clinical staging, treatment modalities, and severity of *Cs* to better characterize these associations. We also acknowledge that our oxWGBS analysis did not detect significant differences in 5-hydroxymethylcytosine (5hmC) levels between *Cs*^+^ and *Cs*^−^ HCC tissues. This null result should be interpreted cautiously. Biologically, 5hmC may exhibit context-dependent dynamics, with limited stability under infection- or stress-related conditions. Technically, oxWGBS resolution can be influenced by DNA integrity, coverage depth, and sample heterogeneity. Future studies incorporating cell-type-specific or single-cell 5hmC mapping may better elucidate hydroxymethylation’s role in parasite-associated HCC. Additionally, the lack of direct evidence of lactylation events in *Cs*-infected HCC restricts our ability to confirm the functional relevance of lactylation in tumor progression. In the future, more direct evidence is needed to prove the causal role of lactylation in HCC progression. Future studies using *Cs*^+^ HCC animal models are crucial to establish the causal role of lactylation in HCC progression and assess its therapeutic potential. Moreover, identifying lactylated proteins and their functional consequences will provide deeper insights into the biological impact of this modification. Future research should integrate high-resolution multi-omics analyses, immunological profiling, and experimental validation to uncover the dynamic interplay of lactylation-dependent epigenetic regulation. These efforts will help identify novel molecular pathways driving tumor progression and may lead to the development of targeted therapies, especially in the context of parasite-related hepatic malignancies, where standard treatments often fail to provide durable responses.

## Conclusion

In summary, we utilized multi-omics data to demonstrate that *Cs* infection disrupts lactylation in HCC. Numerous genes, chromatin open regions, and methylation sites associated with lactate metabolism were identified, some of which contribute to poor prognosis in HCC. Ultimately, this work not only enhances our understanding of lactylation in oncogenesis but also paves the way for innovative strategies to improve patient outcomes in this challenging clinical context.

## Supplementary Information

Supplementary material 1.

Supplementary material 2.

## Data Availability

Data supporting the main conclusions of this study are included in the manuscript.
