# Supplementary material for: Multi-omics analysis of lactate metabolism gene regulation in Clonorchis sinensis-associated hepatocellular carcinoma
Source: Parasit Vectors. 2025 Jul 27;18:301. doi: 10.1186/s13071-025-06947-0 (PMC12302829; doi:10.1186/s13071-025-06947-0)
Supplement: Supplementary file 1 — Supplementary material 1. [file 13071_2025_6947_MOESM1_ESM.docx]

**Supplymentary figures**


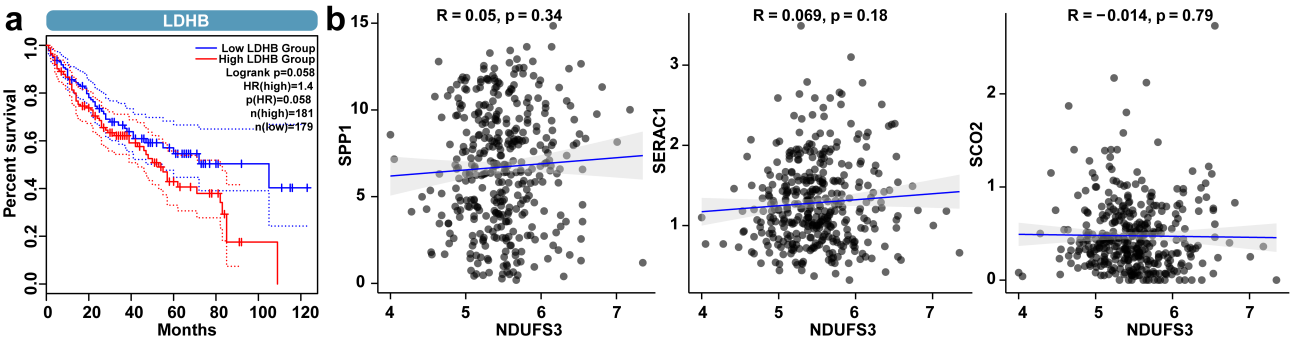


**Fig. S1.** **RNA-seq analysis of LMRGs between *Cs*^+^ HCC tumors and *Cs*^-^ HCC tumors. a.** The Kaplan–Meier curves shows the lack of significant association between the expression levels of differentially expressed LMRGs and survival outcomes in the TCGA-LIHC cohort between *Cs*^+^ HCC tumors and *Cs*^-^ HCC tumors. **b.** The linear plot shows the lack of significant linear correlation among 3 differentially expressed LMRGs between *Cs*^+^ HCC tumors and *Cs*^-^ HCC tumors


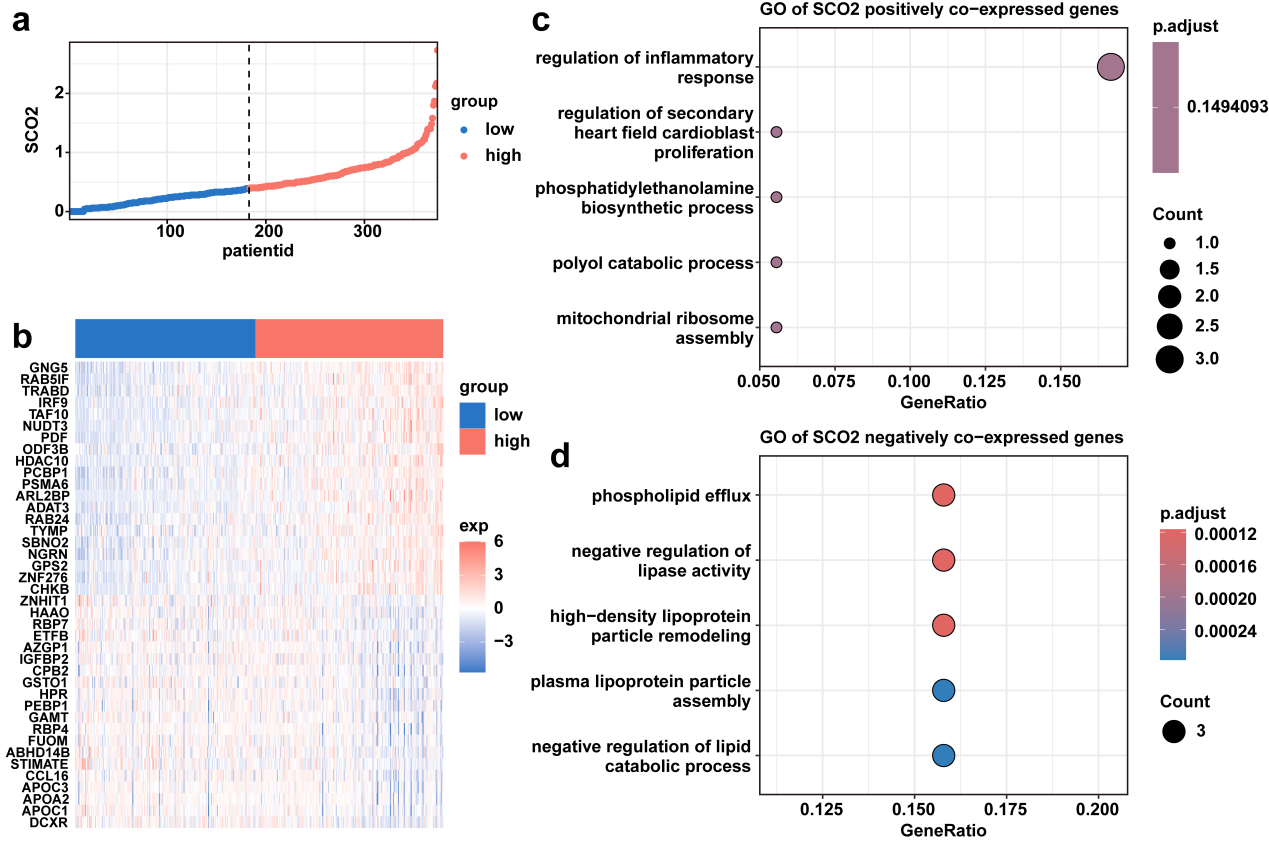


**Fig. S2. Co-expression Analysis of SCO2. a.** Expression levels of SCO2 in HCC patients in the SCO2-low and SCO2-high groups. **b.** Heatmap showing the expression of SCO2 co-expressed genes in the SCO2-low and SCO2-high groups. **c.** GO analysis of genes positively co-expressed with SCO2. **d.** GO analysis of genes negatively co-expressed with SCO2.


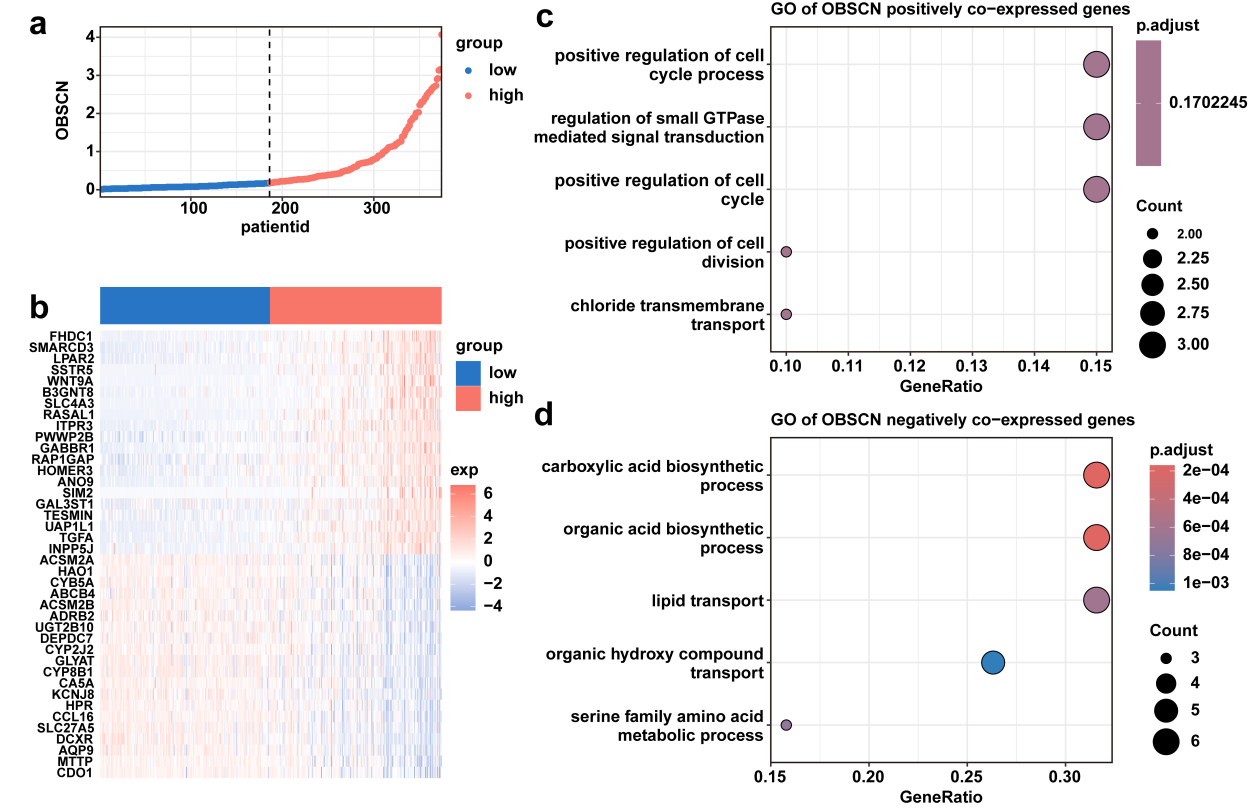


**Fig. S3. Co-expression Analysis of OBSCN. a.** Expression levels of OBSCN in HCC patients in the OBSCN-low and OBSCN-high groups. **b.** Heatmap showing the expression of OBSCN co-expressed genes in the OBSCN-low and OBSCN-high groups. **c.** GO analysis of genes positively co-expressed with OBSCN. **d.** GO analysis of genes negatively co-expressed with OBSCN.


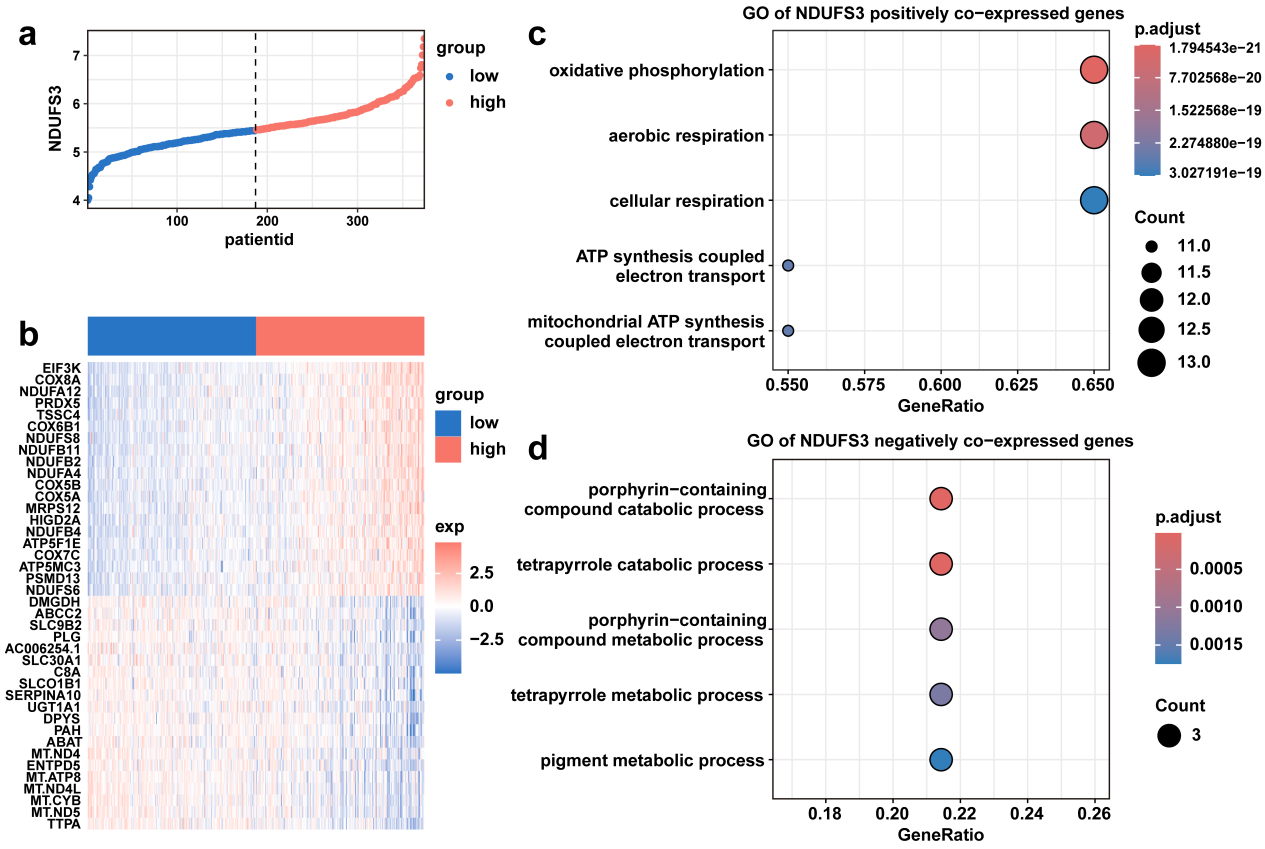


**Fig. S4. Co-expression Analysis of NDUFS3.**

**a.** Expression levels of NDUFS3 in HCC patients in the NDUFS3-low and NDUFS3-high groups.

**b.** Heatmap showing the expression of NDUFS3 co-expressed genes in the NDUFS3-low and NDUFS3-high groups. **c.** GO analysis of genes positively co-expressed with NDUFS3. **d.** GO analysis of genes negatively co-expressed with NDUFS3.


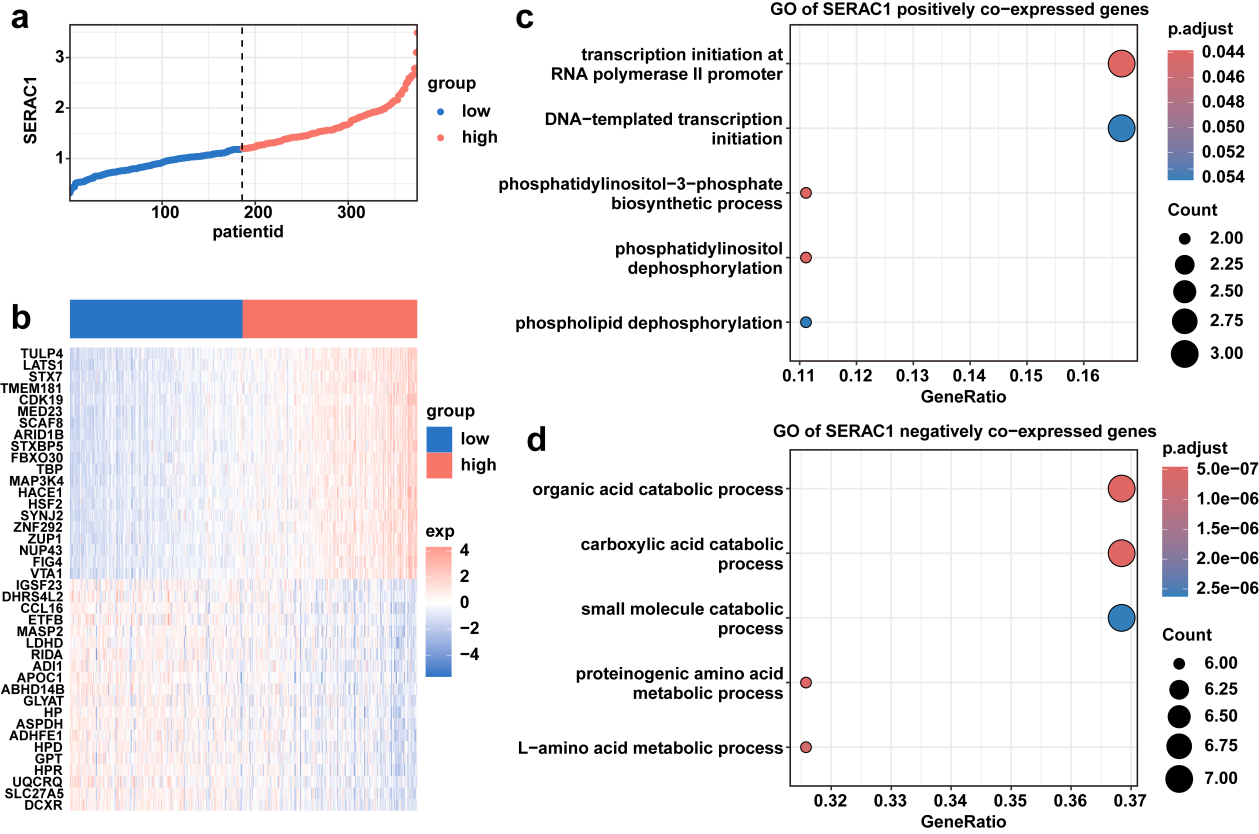


**Fig. S5. Co-expression Analysis of SERAC1.** **a.** Expression levels of SERAC1 in HCC patients in the SERAC1-low and SERAC1-high groups. **b.** Heatmap showing the expression of SERAC1 co-expressed genes in the SERAC1-low and SERAC1-high groups. **c.** GO analysis of genes positively co-expressed with SERAC1. **d.** GO analysis of genes negatively co-expressed with SERAC1.


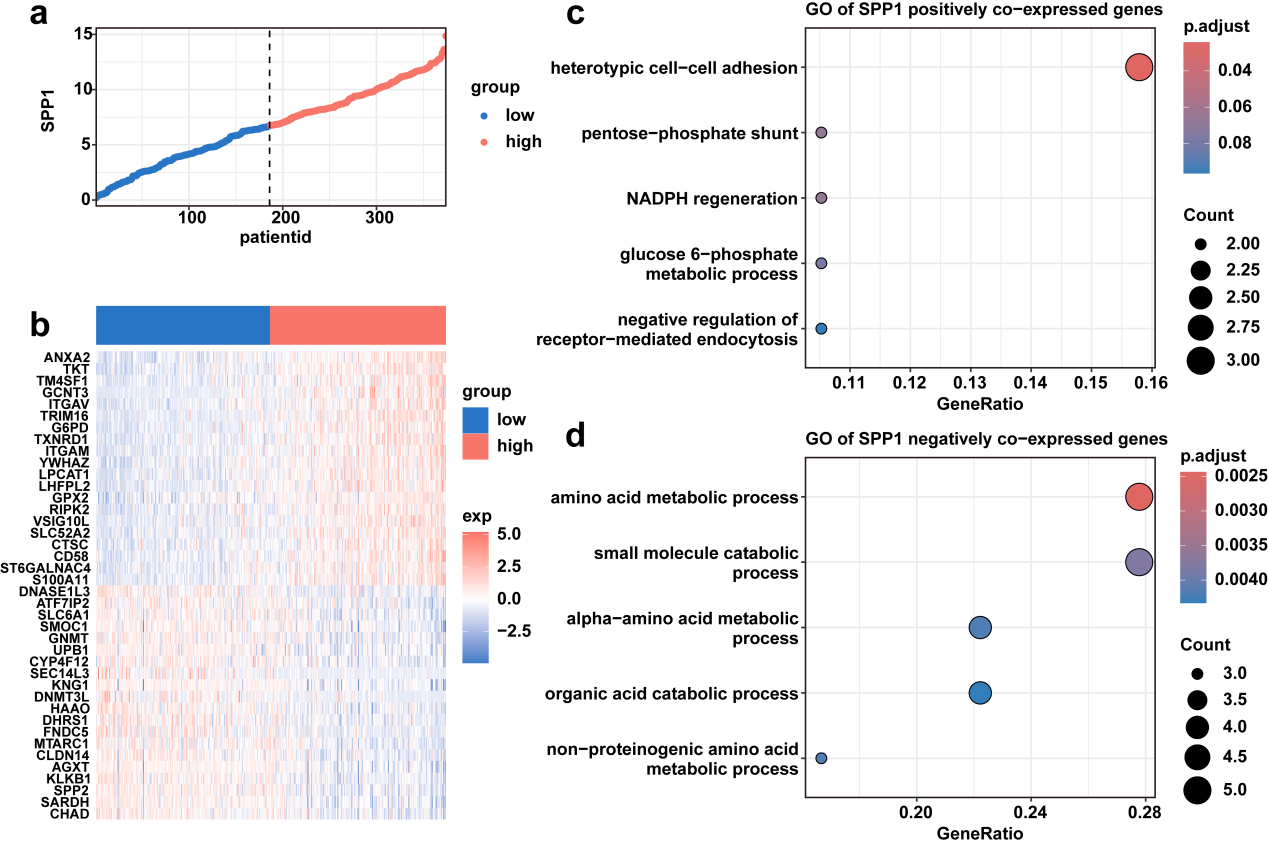


**Fig. S6. Co-expression Analysis of SPP1. a.** Expression levels of SPP1 in HCC patients in the SPP1-low and SPP1-high groups. **b.** Heatmap showing the expression of SPP1 co-expressed genes in the SPP1-low and SPP1-high groups. c. GO analysis of genes positively co-expressed with SPP1. **d.** GO analysis of genes negatively co-expressed with SPP1.
